# Structural Correlates of Rotavirus Cell Entry

**DOI:** 10.1371/journal.ppat.1004355

**Published:** 2014-09-11

**Authors:** Aliaa H. Abdelhakim, Eric N. Salgado, Xiaofeng Fu, Mithun Pasham, Daniela Nicastro, Tomas Kirchhausen, Stephen C. Harrison

**Affiliations:** 1 Laboratory of Molecular Medicine, Boston Children's Hospital and Harvard Medical School, Boston, Massachusetts, United States of America; 2 Rosenstiel Basic Medical Sciences Research Center, Brandeis University, Waltham, Massachusetts, United States of America; 3 Program in Cellular and Molecular Medicine, Boston Children's Hospital and Harvard Medical School, Boston, Massachusetts, United States of America; 4 Howard Hughes Medical Institute, Boston, Massachusetts, United States of America; National Institutes of Health, United States of America

## Abstract

Cell entry by non-enveloped viruses requires translocation into the cytosol of a macromolecular complex—for double-strand RNA viruses, a complete subviral particle. We have used live-cell fluorescence imaging to follow rotavirus entry and penetration into the cytosol of its ∼700 Å inner capsid particle (“double-layered particle”, DLP). We label with distinct fluorescent tags the DLP and each of the two outer-layer proteins and track the fates of each species as the particles bind and enter BSC-1 cells. Virions attach to their glycolipid receptors in the host cell membrane and rapidly become inaccessible to externally added agents; most particles that release their DLP into the cytosol have done so by ∼10 minutes, as detected by rapid diffusional motion of the DLP away from residual outer-layer proteins. Electron microscopy shows images of particles at various stages of engulfment into tightly fitting membrane invaginations, consistent with the interpretation that rotavirus particles drive their own uptake. Electron cryotomography of membrane-bound virions also shows closely wrapped membrane. Combined with high resolution structural information about the viral components, these observations suggest a molecular model for membrane disruption and DLP penetration.

## Author Summary

Non-enveloped viruses (viruses lacking a lipid-bilayer membrane) require local disruption of a cellular membrane to gain access to the cell interior and thereby initiate infection. Most double-strand RNA viruses have an outer protein layer that mediates this entry step and an inner-capsid particle that transcribes their segmented dsRNA genomes and extrudes the capped mRNAs into the cytosol. Removing the two rotavirus outer-layer proteins inactivates the virus, but recoating with recombinant outer-layer proteins restores infectivity. We have labeled the recombinant proteins with distinct fluorophores and the stripped inner-capsid particle with a third fluorophore and reconstituted fully infectious particles from the labeled components. We have followed by live-cell imaging the binding and engulfment of the labeled particles and studied the kinetics of inner-capsid particle release. We have interpreted these events in structural terms by examining images of entering particles from conventional electron microscopy and electron cryotomography. When analyzed in view of our previously determined high resolution structures of the virus particle and its constituents, and of information about conformational changes in the outer-layer components, our data lead to a molecular description of the observed entry steps and of the mechanism of membrane disruption.

## Introduction

Non-enveloped viruses must breach a membrane to enter and infect a cell. Various groups of viruses have evolved distinct molecular mechanisms to carry out this penetration step, which leads to translocation of the infecting particle from an endocytic vesicle or other intracellular compartment to the surrounding cytosol. For example, picornaviruses and reoviruses release a myristoylated peptide, which forms pores in a lipid bilayer [1]. Structural and mutational evidence suggests that rotaviruses penetrate by disruption of an endocytic membrane, driven by a conformational change in one of its outer-surface proteins; the transition has some similarities to the fusion-promoting conformational change of certain enveloped-virus glycoproteins [2], [3]. In no case, however, do we yet have a detailed description of conformational changes in viral proteins couple to changes in the membrane being disrupted nor can we confidently place these events in the context of an intracellular compartment.

**Figure 1:**
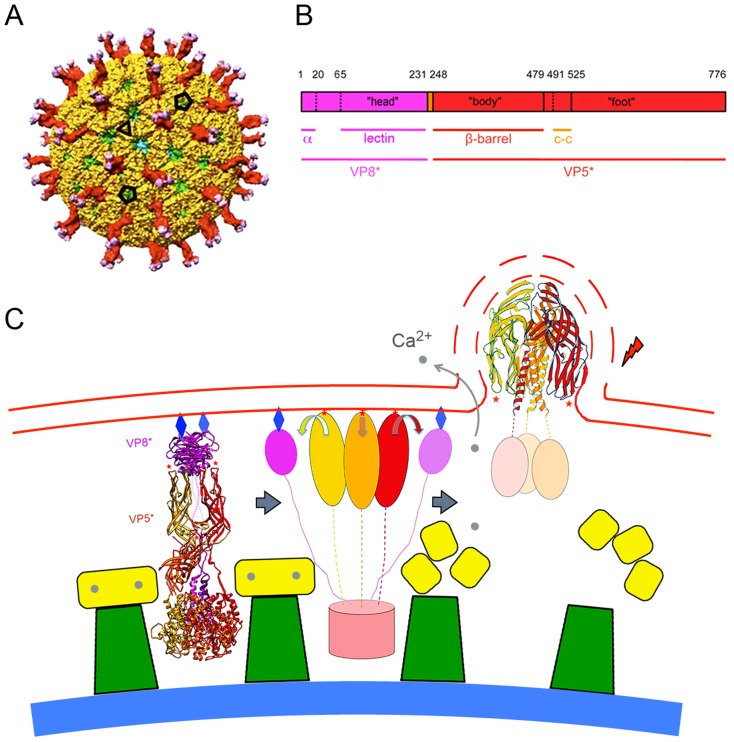
The rotavirus particle and conformational transformations during entry. **A**. Surface representation of the mature rotavirus TLP [9]. VP4 is in magenta (VP8*) and red (VP5*); VP7, in yellow; VP6 (from underlying DLP), in green; VP2 (beneath VP6 and visible just through the opening directly facing viewer), in blue. The projecting part of the VP4 spike is twofold clustered, but the base (buried beneath VP7 in this surface view) is a threefold symmetric trimer. Compare the ribbon diagram on the left in panel. **C**. Scale bar = 100 Å. **B**. Domains of VP4. Trypsin cleavage generates VP8* (magenta) and VP5* (red), by excising residues 231–248 from two of the subunits in a trimer and residues 27–248 from the third. Refer to the left-hand image in **C** for a three dimensional ribbon representation of the mature trimer. “Head” is the receptor-binding, lectin domain of VP8*; “body”, the β-barrel domain of VP5*, with hydrophobic loops at its tip; “foot”, the largely α-helical domain clamped beneath VP7 in the TLP. Numbers above the bar refer to amino-acid residues; lines immediately beneath the bar, substructures of the protein (α: N-terminal helix, anchors VP8* into the foot; lectin: receptor-binding domain of VP8*; β-barrel: projecting domain of VP5*; c-c: coiled-coil structure that forms when VP5* folds back, as in the right-hand image in **C**). **C**. Proposed correlation of conformational changes in the mature VP4 spike with remodeling and breaking of a bilayer membrane. VP7 (yellow), VP6 (green), and VP2 (blue shell) are shown schematically. ***Left***: mature spike, anchored into the DLP by VP7, a Ca^2+^-stabilized trimer. VP4 is activated by cleavage to VP8* (magenta ribbons) and VP5* (red, orange and gold ribbons). Red stars: hydrophobic loops of VP5* β-barrel; blue diamonds: glycolipid head groups to which the lectin domains of VP8* attach. ***Center***: Schematic diagram of the product of an initial conformational change, in which VP8* lectin domains dissociate from their positions covering the hydrophobic loops of VP5*, exposing them for interaction with the membrane. VP8* lectin domain is simplified here to a magenta oval; VP5*, to red, orange, and gold ovals (β-barrels) connected by flexible links to the foot (light red cylinder). ***Right***: loss of Ca^2+^ causes VP7 (yellow) to dissociate from DLP, releasing VP5*, which is now free to fold back to its most stable, postcleavage conformation. Interaction between membrane and VP5* hydrophobic loops (red stars) couples membrane distortion and rupture (dashed lines) to folding back of VP5*. See [2], [9] and text of this paper for further structural details.

The double-stranded RNA (dsRNA) viruses offer particular advantages for analyzing cell entry by individual virions. An infectious rotavirus particle encapsidates eleven distinct double-stranded RNA segments within a three-layer protein coat (a “triple-layered particle”, or TLP: Fig. 1A) [4]. The inner two layers, composed of viral proteins 2 and 6 (VP2 and VP6) respectively, remain associated with the RNA as a “double layered particle” (DLP), even after penetration. The outer layer, composed of two proteins, VP4 and VP7, is the agent that delivers the DLP into the cytosol. VP7, a Ca^2+^-stabilized trimer [5], clamps onto the VP6 trimers [6], [7], which form a T = 13 icosahedral array on the DLP surface [8]. The VP7 lattice thus generated holds the sixty trimeric VP4 “spikes” in place [9]. Tryptic cleavage of VP4 into two fragments, VP8* and VP5*, is an activation step that primes the protein for conformational changes linked to penetration (Fig. 1B) [10]–[13]. The final, stable conformation of VP5* is probably the folded-back structure shown in the last panel of Fig. 1C, which illustrates a model for membrane disruption generated by conformational changes in VP5*. The model derives from structural studies of VP4 and its fragments [2], [14]–[16]; the experiments we describe here test some of the model's predictions.

Rotaviruses infect enterocytes of the small intestine; in culture, they grow in a variety of epithelial-origin cells [4]. For the best characterized strains, attachment and internalization depend on a surface glycan. Animal rotaviruses generally require sialic acid, either at a terminal or subterminal position (the corresponding viruses then being neuraminidase sensitive or insensitive, respectively [17]), but some human rotaviruses do not [4]. Recent work shows that one such virus interacts with a non-sialylated glycan, the A-type histo-blood group antigen, which contacts VP8* at the position usually occupied by sialic acid [18].

The glycans identified as rotavirus receptors probably mediate uptake when they are headgroups of glycolipids. Knockdown with RNAi of enzymes required for ganglioside biosynthesis in the MA104 epithelial cell line reduced infectivity of several different human and animal rotaviruses, all of which bind sialic acid [19]. The same viruses appeared to attach adequately to the surface of the RNAi-treated cells, but failed to penetrate; knockdown of ganglioside biosynthesis would not have affected the presence of similar glycans on glyco*proteins*, which therefore could have been sites of the observed non-productive attachment. Conflicting evidence concerning the role of particular routes of uptake in rotavirus infection may come from the potential for more than one productive pathway of entry and from the choice of a particular preferred pathway by any specific viral strain. Thus, one paper reports that of four strains tested in MA104 cells, three showed a dependence on components of clathrin-mediated endocytosis, while the fourth (RRV, the one we have used for the experiments in this paper) did not [20].

Previous efforts to detect entering virions directly by optical microscopy have relied on immunofluorescent staining with antibodies specific for specific conformational states of VP4 (or VP8* and VP5*), VP7, and VP6 [21]. Use of fixed and permeabilized cells, as required for immunofluorescence, precludes tracking of individual virions, and kinetic inferences are therefore indirect. The functional recoating procedure that allows us to add back recombinant VP4 and VP7 to DLPs stripped of their outer layer [22] provides an effective means to label each component with a distinct fluorophore and then to follow entry by live-cell imaging. We report in this paper a series of such experiments, from which we derive the following conclusions. Virions bind tightly upon contact with a cell, becoming relatively immobile on the cell surface in less than a minute. Within about five minutes of attachment, many of the fluorescently tagged rotavirus particles have become sufficiently engulfed that they are inaccessible to antibodies and insensitive to elution with EDTA. Clathrin or the AP-2 clathrin adaptor colocalize only rarely with entering particles, and infectivity in these cells is dynamin independent. Most particles that release a DLP into the cytosol – an event marked by separation of the DLP label from the labels on VP7 and VP4 – have done so by ten minutes post attachment. At later time points, we find virions in Rab5-labeled early endosomes, but these particles rarely penetrate. Moreover, we find that infectivity is insensitive to overexpression of Rab5 mutants. Images from thin-section electron microscopy (EM) and electron cryotomography (cryoET) are consistent with these live-cell observations and suggest that direct contacts between virion and membrane drive engulfment.

## Results

### Fluorescent labeling of individual virion components

**Figure 2:**
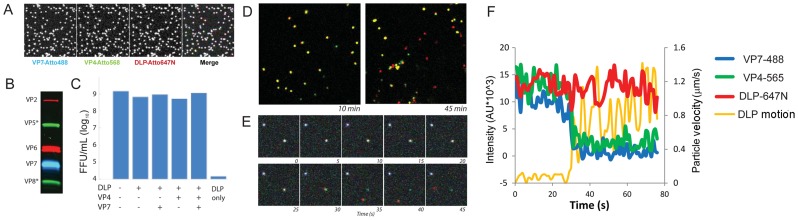
Fluorescent labeling of recoated TLP. **A**. Images of particles, adsorbed to coverslip, at each of three wavelengths and overlay. **B**. Analysis of recoated, purified TLPs by 12% SDS-PAGE. **C**. Fluorescent focus assay measuring effect of labeling on reacoated particle infectivity, with labeling as shown. **D**. Images of doubly labeled (VP7, green; DLP, red) recoated particles, 10 min and 45 min following addition to BSC-1 cells. Uncoated particles (red) are evident at the later time point. **E**. Time lapse sequence of triply labeled particles uncoating in BSC-1 cells. Time frames at 5 sec intervals from an arbitrary time point following addition of particles to cell. Pseudo-colors: VP7, blue; VP4, green; DLP, red. **F**. Graphic representation of time lapse sequence in panel E; images recorded at 1 sec intervals. Intensities (colors as in E) evaluated in a box moving with the DLP; orange curve: velocity of DLP.

We can monitor individual behaviors and roles of the DLP, VP4 and VP7 during rotavirus infection by combining previously optimized recoating techniques [22], [23] with amine-specific, fluorescent labeling of these components (Materials and Methods). We produced reconstituted rhesus rotavirus (RRV) TLPs with two (“doubly-labeled”) or three (“triply-labeled”) structural components linked to spectrally distinct fluorescent dyes, which were discernible in confocal microscopy or upon electrophoresis (Figs. 2A,B). The modifications had no effect on viral infectivity, as assessed by fluorescent focus assays (Fig. 2C). RRV requires cell-surface sialic acid for attachment and subsequent infection. We verified that our labeled, assembled particles had the same requirements when infecting the BSC-1 cells used here, by comparing virion binding to neuraminidase treated and untreated cells. The results (Fig. S1) confirmed that removal of sialic acid from surface glycans greatly reduces viral attachment.

### DLP release

**Figure 3:**
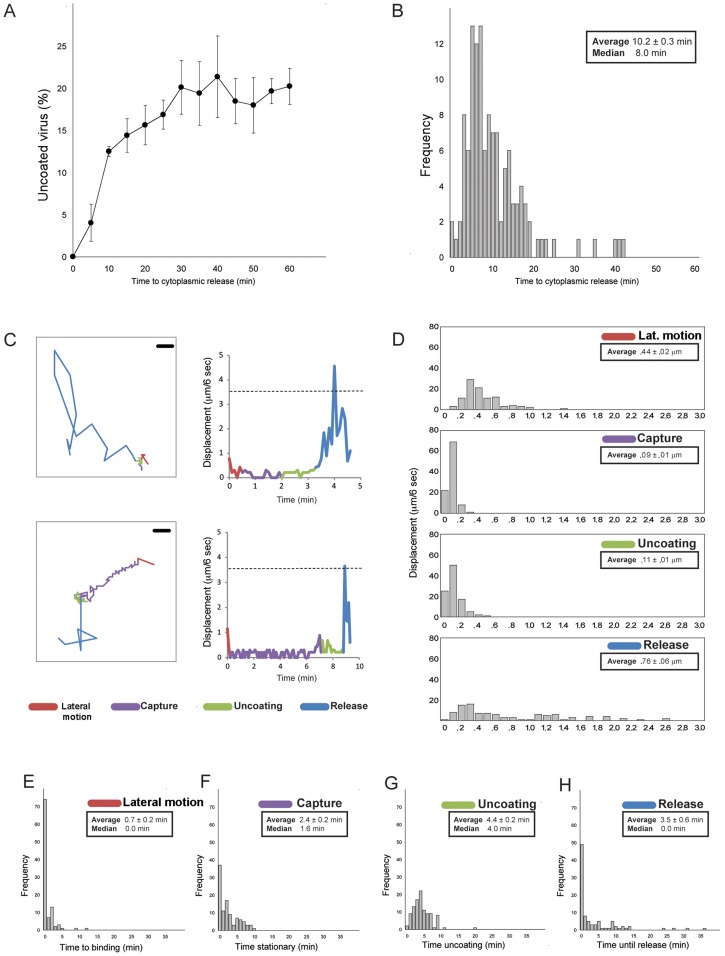
Statistics of entry and uncoating. **A**. Percent of doubly labeled TLPs (labels on DLP and VP7) that have uncoated, as a function of time after addition of pulse of virus to BSC-1 cells. (Bolus of virus added, excess removed and replaced with fresh medium). Images recorded at 6-sec intervals. Average of three experiments; bars show standard errors. **B**. Histogram of interval between binding and uncoating; images recorded at 6-sec intervals. **C**. Detailed traces for two individual particles. Four stages color coded: lateral motion (red), capture (purple), uncoating (green), and DLP release (blue). Left-hand panels show projected particle positions (scale bar = 1 µm); right-hand panels, particle displacement for each 6-sec interval between successive frames. The dashed line shows the expected displacement in 6 sec for a particle of radius 350 Å undergoing Brownian motion in a medium of effective viscosity 10 times that of water at 37°C, as estimated from the particle-radius dependence of cytoplasmic viscosity for cells in culture [48]. **D**. Distribution of projected 6-sec displacements of rotavirus particles during each phase; one hundred particles analyzed for each panel. **E–H**. Time (in minutes) spent in each phase, for one hundred particles. Images recorded at 6-sec intervals.

To visualize rotavirus cell entry in vivo, we infected BSC-1 cells with reconstituted, labeled virus; we then imaged the course of infection by spinning-disk confocal microscopy. BSC-1 cells, which are permissive for various rotaviruses [24], spread on the coverslip to generate thin, broad periphery, placing much of the non-adherent cell surface within the focal depth of the microscope and allowing us to capture in one image a large area of plasma membrane. We added doubly- or triply- labeled virus to cells and followed over time the fate of the various components. Over a 10–30 min period after addition of labeled virions, we observed gradual accumulation in the cytoplasm of rapidly moving, singly labeled particles, corresponding to dye-linked DLPs – i.e., virus particles that have lost their outer coat (Fig. 2D). Careful tracking of individual particles revealed a loss in the intensity of the outer-layer proteins preceding an abrupt separation of DLP label from residual outer-layer protein label and rapid diffusional motion of the DLP away from the position of initial attachment (Fig. 2E,F). We interpret this event as release of the DLP into the cytosol. In some cases, the rapidly moving DLP left behind a distinct residue of VP7 and VP4 fluorescence close to the plasma membrane (Fig. 2E); in the majority of cases, the fluorescence intensity associated with the outer coat of the virus had decreased to the threshold of detection by the time of DLP release. The motion of the released DLP was random and much faster than that of TLP from which it derived (Fig. 2E,F; Fig. 3C,D) and consistent with free diffusion in the cytosol for a particle of radius ∼350 Å (Fig. 3C,D; see caption to Fig. 3C). By 30 minutes after addition, the fraction of released particles had reached a plateau of about 20% (Fig. 3A). (In principle, our observations on DLP release do not exclude the possibility that the DLP has “budded away” from the other components, retaining some surrounding membrane. There is no clear molecular basis for such a mechanism, however: VP5* binds membranes, and DLPs do not. The properties of VP4 mutants make an interpretation other than access to the cytosol very unlikely – see below.)

### Time course of cell entry

The distribution of times to DLP release in the experiments we analyzed is shown in Figure 3B. The mean time from binding to penetration is approximately 10 minutes; the shortest times are between 6 and 8 minutes.

Upon binding to the cell surface, some particles appeared to move about relatively rapidly on the cell surface (>4 µm/min: “lateral motion”), before becoming nearly stationary (<1 µm/min: “capture”); the majority (>70%) appeared to not to have a lateral-motion phase at all. A correlated, centripetal motion of captured particles in a given region of the cell surface probably arose from retrograde flow of the plasma membrane on which they were bound (e.g., capture phase in Fig. 2C). For the subset of captured virus particles that ultimately uncoated, the fluorescence intensity of the labeled outer-layer protein(s) VP7 and/or VP4 often slowly declined, while the particles remained more or less fixed. The event we designate as “release” is characterized by the sudden onset of rapid motion of the uncoated DLP (average speed >7 µm/min, with frequent change of direction), leaving in place any residual fluorescence from outer-layer proteins.

Figs. 3E and 3H show distributions for the durations of the various phases of entry just described. For the majority of viruses that had a lateral-motion phase (<30% of the total), capture occurred within 5 minutes; the mean duration of this phase for all particles was less than a minute (∼0.7 minutes). The mean interval between capture and onset of uncoating (decline of fluorescence intensity for VP4 and/or VP7) was ∼2.5 minutes, and the mean duration of the “uncoating” phase (time between onset of uncoating and decline either to undetectable VP4 or VP7 signal or DLP release, whichever came first) was ∼4.5 minutes. For about half the particles (∼50% frequency), release coincided with the end of apparent uncoating; for the remaining particles, the DLP appeared to be relatively stationary after the outer-layer fluorescence had declined maximally. For all particles, the mean interval between loss of detectable VP4 or VP7 and release of the DLP was ∼3.5 minutes. During this time, there may have been some outer-layer protein continuing to dissociate from these particles, but with a total fluorescence below the detection threshold of our microscope configuration.

### DLPs are released from compartments sequestered from the external medium

**Figure 4:**
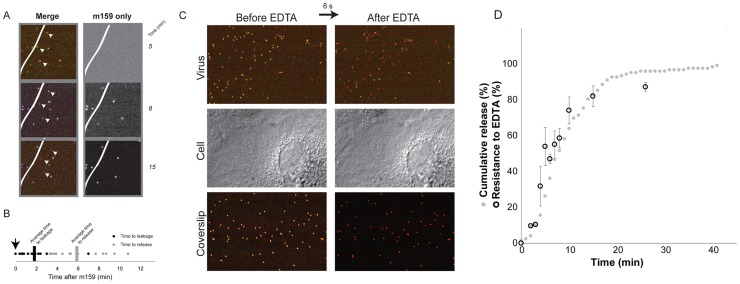
Internalization. **A**. Image of three particles that uncoat after addition of fluorescently labeled m159 antibody to the medium. (Virus pre-bound to cells, excess medium withdrawn, and antibody-containing medium then added.) Particles that uncoat do not bind antibody. White line represents edge of cell; particles on the coverslip all bind antibody. **B**. Time to initiation of uncoating (decrease in VP7 and VP4 label intensities) of particles that do not bind antibody m159. The antibody was added 5–7 minutes after addition of virus to cells, as indicated by the arrow; images were collected every 6 seconds. **C**. EDTA pulse. Top panel: doubly labeled virus bound to cell, before and after EDTA pulse. Middle panel: DIC images of the same cell; Bottom panel: particles bound to coverslip. **D**. Percent of virus resistant to EDTA uncoating, as a function of time between addition of virus and pulse of EDTA (black open circles) and cumulative representation of data in 2B (gray solid circles).

The VP7-directed neutralizing antibody, m159, inhibits rotavirus entry by preventing uncoating of trimeric VP7 [25]. We used fluorescently labeled m159 to determine when in the time course of entry just described the virion becomes inaccessible (Fig. 4A). We added antibody 5–7 minutes after adding virus. Some cell-attached virions bound the antibody, but a number failed to do so. Many in the latter group proceeded to uncoat and release, with the characteristic sequence of stages described above (Fig. 4A). We measured the intervals between addition of antibody and onset of uncoating and time DLP release. The mean time between antibody addition and the onset of uncoating of antibody-inaccessible particles was about 2 minutes, with a range between 0 and ∼7 minutes; release followed by about 4 minutes the onset of uncoating (Fig. 4B). Virus particles exposed to the medium on the surface of the cell bound antibody within seconds of its addition and never uncoated (data not shown), in keeping with the known characteristics of m159; controls with particles bound to the coverslip of the imaging chamber confirmed that all viruses that are exposed to the medium bind the antibody quickly and efficiently (Fig. 3A and data not shown).

### Kinetics of internalization

Chelation of Ca^2+^, by EDTA or similar agent, dissociates the VP7 trimer and strips the outer layer from TLPs [5]. When cells are pulsed with EDTA-containing medium, any virus not internalized loses both VP7 and VP4 and dissociates from the cell surface (Fig. 4C, top panel). We exposed the cells to EDTA-containing medium for no longer than 10 seconds before reintroduction of medium containing calcium, thus minimizing damage to the cells (Fig. 4C, middle panel). As a control, viruses that were bound directly to the coverslip were monitored to verify that all viruses exposed to medium had lost their outer layer (Fig. 4C, bottom panel). By about 5 minutes, ∼50% of cell-bound viruses were EDTA resistant (Fig. 4D), in good agreement with the time between attachment and the onset of uncoating. Comparison of the overall time to uncoating (Fig. 3B) with the kinetics of internalization as determined by EDTA resistance indicates that in our experiments there was on average a ∼1–3 minute lag between time to internalization and time to cytoplasmic release. Thus, sequestered virions spend only a relatively short time in an uptake compartment before release of the DLP into the cytosol.

### Effects of mutations in VP4 and VP7

**Figure 5:**
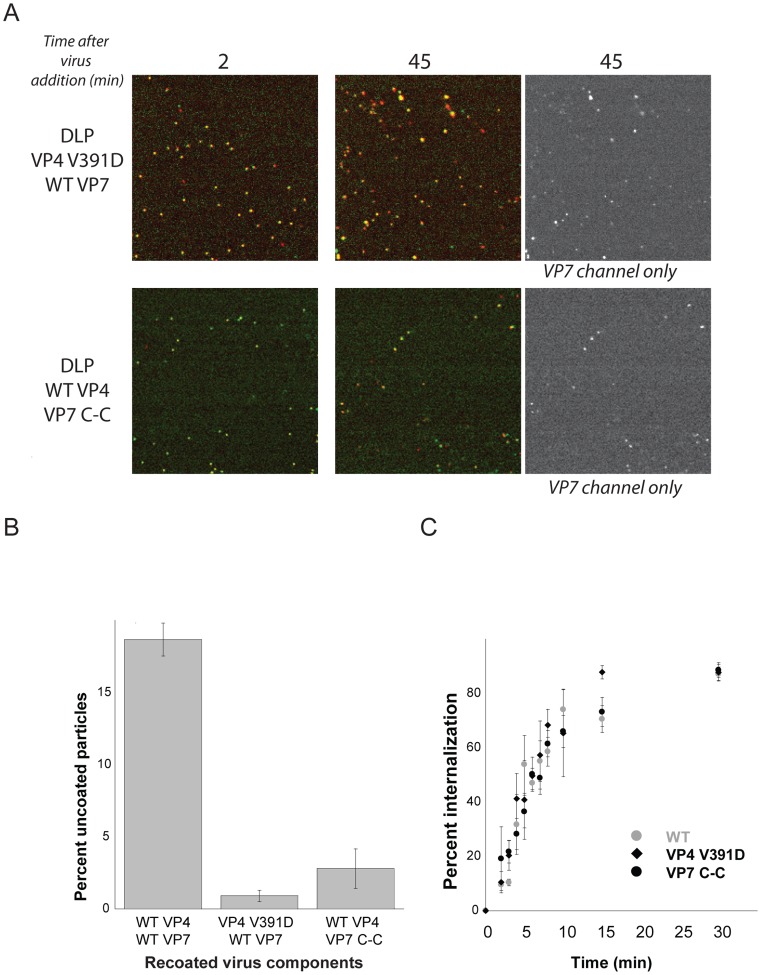
VP4 fusion-loop mutant and VP7 disulfide-linked trimer. **A**. Effects of mutations on uncoating. Particles recoated with VP4 fusion-loop mutant (top panel) and VP7 disulfide-linker trimer (bottom panel) added to BSC-1 cells and imaged at 2 min and 45 min. All particles in the field at 45 sec retain VP7. **B**. Percent uncoating for particles recoated with wt proteins, with VP4 mutant and VP7 wt, and with VP4 wt and VP7 mutant. Average of three experiments; bars show standard error. **C**. Percent of particles protected from EDTA dissociation (for wt and VP4-391D) and from access by m159 (for VP7 C-C) as a function of time. Average of three experiments; bars show standard error.

Previously characterized mutations in the hydrophobic loops of VP5* block infection but do not interfere with binding or uptake [3]. These same mutations also prevent trimeric VP5* from associating with membranes, when the trimer is prepared *in vitro* and in the presence of liposomes, by successive treatment with chymotrypsin and trypsin (which generates the species designated “VP5CT”: [26]). They also fail to release α-sarcin into the cytosol [3]. We followed the uptake of particles recoated with one of these VP4 mutants, V391D, which reduces infectivity by a factor of about 10^4^ [3]. The data in Fig. 5 show that although these particles acquire EDTA resistance with normal kinetics, they fail to release DLPs, even after 45 minutes. Combining this observation with loss of α-sarcin release by the same mutant strengthens our conclusion that DLP release corresponds to cytosolic access. The particles recoated with mutant VP4 also exhibit a somewhat longer lateral motion phase than do wild-type TLPs (Fig. S2), suggesting that the hydrophobic loops might participate in engulfment as well as in membrane disruption.

Disulfide crosslinking of VP7 trimers also blocks infection, by preventing VP7 dissociation [25]. Particles recoated with this modified VP7 have normal uptake kinetics, which we have followed by accessibility to the m159 antibody (data not shown), but like the VP4 V391D mutation, the VP7 disulfide crosslinks prevent DLP release (Fig. 4). Thus, the effects of these mutations on infectivity correlate closely with their effects on the entry pathway detected by live-cell imaging.

### Entry pathways

**Figure 6:**
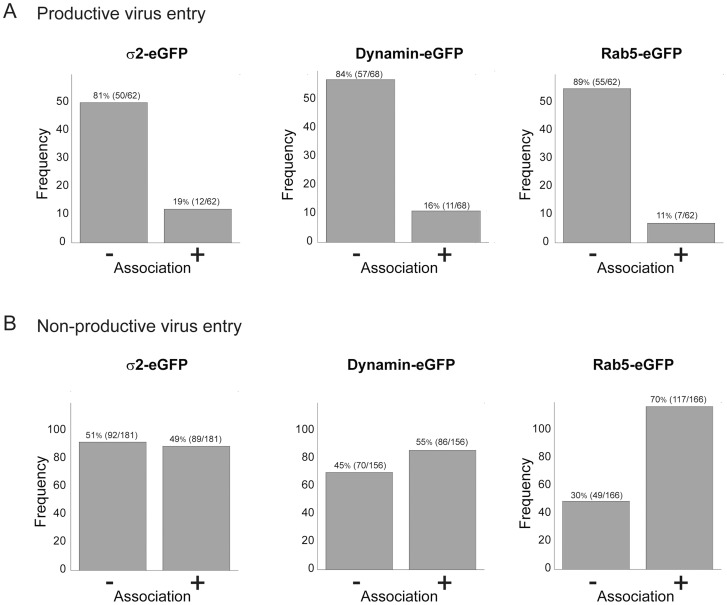
Association of productive (A) and non-productive (B) particles with AP-2 clathrin adaptor, dynamin, and Rab5. “Productive particles” are those that bind and uncoat as in Figure 3; “non-productive particles”, those that fail to uncoat within 30 mins of binding. Of 62 productive particles followed in cells transfected with σ2-eGFP (**A**, left), 50 did not colocalize with σ2 at any time, while 12 colocalized with σ2 at an early time point. Likewise, of 62 productive particles followed in Rab5-eGFP transfected cells (**A**, right), only 11 appeared to colocalize with Rab5, while of 166 non-productive particles (**B**, right), a substantial majority ended up in Rab5 endosomes. Cells stably transfected with σ2-eGFP or Rab5-eGFP or transiently transfected with dynamin-eGFP.

Viruses can exploit a variety of cellular uptake mechanisms. We looked for colocalization of entering rotavirus particles with markers for particular intracellular compartments. We followed each particle for long enough that we could confidently classify its entry as “productive” or “non-productive” – the former defined by the release step described above. Less than 20% of the productive events involved particles that had co-localized at any time with the plasma-membrane clathrin adaptor, AP-2, with dynamin (required for budding of clathrin-coated vesicles and probably for caveolin-associated membrane remodeling), or with Rab5, a marker for early endosomes (Fig. 6A). Moreover, non-productive events correlated positively with Rab5 (Fig. 6B). We ruled out more directly a requirement for Rab5 by ectopic expression of two Rab5 mutants – a dominant negative, inactive form (Rab5DN, bearing the mutation S34N) and a constitutively active form (Rab5CA, bearing the mutation Q790L). We transfected BSC-1 cells, seeded on prepared coverslips, with vectors encoding the Rab5 mutants fused to eGFP. One day later, we added RRV at various MOI, allowed infection to proceed overnight, fixed the cells, and assayed by immunofluorescence for VP7 expression. Each coverslip, corresponding to a particular mutant Rab5 and a particular MOI, had transfected and untransfected cells. We could therefore determine for each MOI the proportion of cells infected, both for those expressing the Rab5 mutant and for those expressing only endogenous, wild-type Rab5. The data in Fig. S3 show that neither of the two mutant Rab5s influenced the efficiency of infection.

We conclude, from the low frequency of escape from Rab5 endosomes and from the negligible effects of Rab5 mutants, that rotavirus entry in BSC-1 cells does not require transport to early endosomes and that particles with a Rab5-endosomal fate may have reached a dead end. This conclusion is consistent with the rapid time course of uncoating and release documented above. We also found that rotavirus infectivity in BSC-1 cells is insensitive to hydroxy-dynasore, a second generation dynamin inhibitor (Fig. S4).

### Electron microscopy of virion uptake

**Figure 7:**
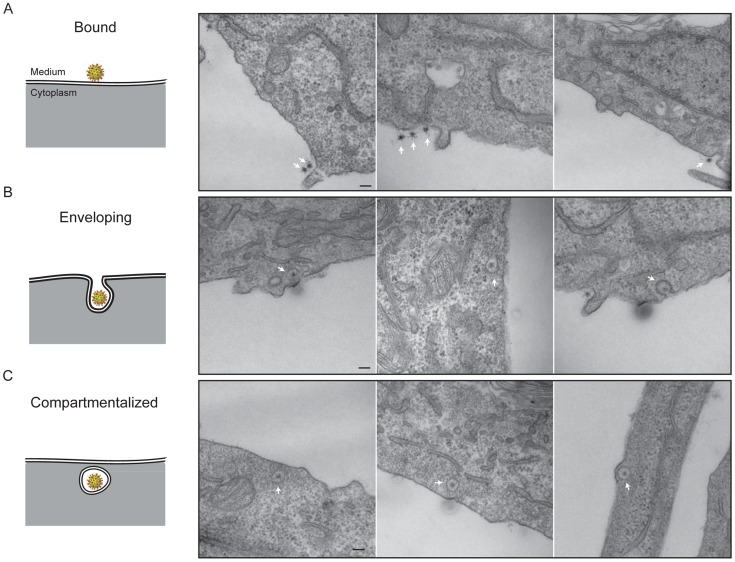
Thin-section electron microscopy: stages of rotavirus entry. Experiments done with authentic virions, not recoated DLPs. **A**. Surface-bound particles. The center of the particle (RNA) takes up stain particularly avidly. **B**. Engulfment phase. In the right-hand panel, the particle may be entering by a clathrin-coated pit. **C**. Compartmentalized virions.

We followed stages of viral entry by conventional thin-section electron microscopy, to complement the live-cell imaging studies with visualization of cellular ultrastructure and membrane morphology. We exposed cells to virus for various times, then chemically fixed the cells and prepared them for EM by standard techniques (see Methods). Fig. 7 shows three apparent stages of viral uptake, which we label “bound”, “engulfing”, and “enclosed”. (We cannot, of course, rule out a residual membrane neck, oriented away from the plane of the section, connecting an “enclosed” particle with the cell surface.) We interpret the first and last of these stages as corresponding, respectively, to the “lateral-motion/capture” and “capture/uncoating” phases detected by live-cell imaging, with engulfment as the transition between them; the asynchrony of events at the cell surface and the times required for fixation prevent any direct experimental correlation of the two methods.

Heavy-metal staining of virions is somewhat irregular, with a strong concentration of stain on the RNA interior and relatively weak and spotty staining of the protein shells. Weak but perceptible bridges between the virus particles and the cell membrane are consistent features of all the images. The most striking property of the engulfing and enclosed particles is the relatively uniform distance – roughly 550 Å – between the surrounding membrane and the center of the virion. There are no evident specializations on the cytosolic side of the membrane, such as membrane-bound molecules or cytoskeletal assemblies (Fig. 7), except for some infrequent examples of clathrin-mediated uptake (Fig. 7B, right-hand panel). The most straightforward interpretation is that the particles are driving their own engulfment through direct contacts with the surrounding membrane. We show in the Discussion that the dimensions and structural properties of the VP4 spikes on infectious TLPs are consistent with this interpretation. The fully enclosed particles are generally within 100–200 nm of the plasma membrane, as measured in the plane of the section, consistent with the relative immobility of sequestered virions up to the time of DLP release.

### CryoET of attached virions

**Figure 8:**
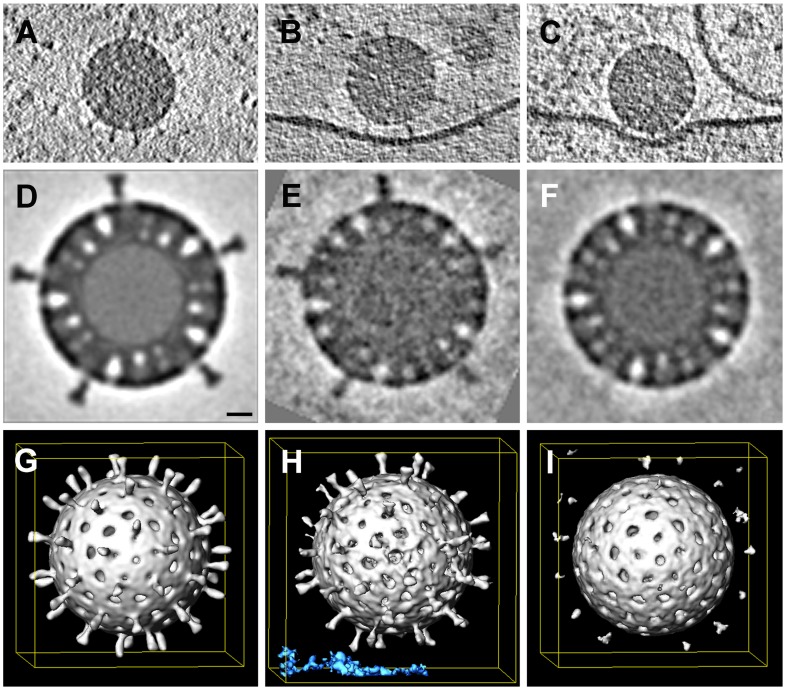
Whole-cell cryo-tomography of surface-bound rotavirus particles. **A–C.** Tomographic slices through individual virions, free (**A**) and bound (**B,C**) to cell membrane. Note differences in spike extension in **B** and **C** (red arrowheads). **D–I.** Tomographic slices (**D–F**) and isosurface renderings (**G–I**) of subtomogram averages of rotavirus particles. Subtomogram averages: (**D,G** & **F,I**) after applying *icosahedral symmetry* (**D,G:** 1080 repeats from 18 virions; **F,I**: 420 repeats from 7 virions); for (**E,H**), we classified the repeats and averaged only those 78 repeats from 5 virions for which spikes were in contact with membrane (blue density).

The periphery of BSC-1 cells is thin enough in some regions to allow recording of a tomographic tilt series of rapidly frozen cells preserved in a nearly native state. We grew BSC-1 cells on carbon-coated, gold grids (see Methods). At 24 hours after depositing the cells on the grid, we added RRV at high concentration to maximize the likelihood of finding attached virus particles and recorded tilt series from positions at which the edge of a cell was thin enough for transmission EM. The high virus concentration often yielded clusters of particles at the cell surface. We concentrated our analysis of cryo-tomograms on isolated, attached or partially engulfed virions, in order to correspond as closely as possible to the events we followed by live-cell imaging, which always had the fluorescence intensities of single recoated TLPs. We detected various states of attachment and engulfment, as illustrated in Fig. 8. Unattached particles showed clearly defined VP4 spikes; icosahedral averaging of 18 virus particles (1080 repeats) produced a tomographic 3D reconstruction with ∼4 nm resolution (Fig. 8D,G). Membrane-attached particles appeared to have induced various degrees of membrane wrapping, with the bilayer at a uniform distance from the surface of the particle. We detected two classes of virion-membrane contacts – those for which the separation of the particle surface from the membrane corresponded to the full extent of the VP4 spike (Fig. 8B) and those for which the separation was substantially smaller (Fig. 8C). We computed subtomogram averages of just the icosahedral repeats facing the membrane. Contacts in the former class had VP4 spikes similar in appearance to those on free virions, but with some indication of disorder at their tips (Fig. 8E, H); contacts in the latter class had largely disordered spikes (Fig. 8F, I), which we infer had undergone a conformational change. We do not yet have enough images to describe this apparent spike reorganization in more detail, but we suggest that it could reflect VP8* dissociation from the tips of the spikes and interaction of the VP5* hydrophobic loops with the membrane bilayer. The well-preserved particles and the uniformity of membrane invaginations around them reinforce our interpretation of the images from conventional EM in Fig. 7.

## Discussion

Functional recoating of rotavirus DLPs with tagged, recombinant VP4 and VP7 enables direct observation, by live-cell imaging, of the stages and kinetics of RRV entry into BSC-1 cells. We have labeled all three components independently with distinct fluorophores without compromising infectivity of the recoated particles and followed them through a reproducible succession of stages leading to DLP release into the cytosol. When added to cells, virions attach and immobilize rapidly, sometimes with an initial short period (<1 min) of lateral motion on the cell surface. Within about 5 minutes of attachment, the particles become inaccessible to EDTA (which releases accessible virions from the cell by dissociating VP7) and to VP7-directed antibody. Abrupt release of the DLP follows within a further 3–5 minutes. The sequestered virions lose VP4 and VP7 at variable rates until DLP release, but any residual VP4 and VP7 at the time of release remain at the site of penetration. The sequence of events leading to DLP release does not require association with clathrin adaptors or dynamin, and under the conditions of our experiments, association with Rab5 endosomes appears to be a dead end. Mutations in VP4 and VP7 that block infectivity prevent DLP release without affecting particle sequestration.

The particle to infectious unit ratio is high for nearly all viruses that infect animal cells, and the low efficiency of infectious outcome can affect interpretation of experiments on mechanisms of entry. We use the phrase “productive entry” here to mean release of the DLP into the cytosol – the step on which we focus – not synthesis and assembly of progeny virions. Various factors influence whether a particle released into the cytosol will then initiate infection. Indeed, we expect the probability of infectious outcome to be low, even for rotavirus particles containing a perfect assortment of eleven genomic segments that have entered by the principal infectious route, because a sequence of potentially inefficient steps follows DLP release. Evasion of host-cell innate defense mechanisms and transport of the released particle to a location in the cell appropriate for viral RNA and protein synthesis are both likely to reduce the chance that a released particle will produce detectable progeny. For example, if each of only two subsequent events were to have the same relatively high efficiency (∼20%) as does DLP release in the experiments reported here, and if the proportion of genetically competent particles and the efficiency of attachment of incubated virus to cells were each 50%, the overall particle to infectious unit ratio would be about 500∶1, consistent with published estimates [27] and with our own estimates for fresh RRV preparations. Thus, the route of penetration with the highest frequency is very likely to be the one taken by any particle that ultimately generates an infectious outcome.

The release events we describe here have all the properties we expect for functional penetration. They are of much higher frequency than any other mode of productive release we can detect by following individual virions; they deliver DLPs to the cytosol, as monitored using distinguishable fluorophores for DLP and outer-layer proteins; they are sensitive to mutations with known structural and functional consequences. In particular, we expect the VP4 hydrophobic-loop mutant, which has greatly impaired infectivity, to be defective in membrane disruption and particle release, because that mutant also fails to mediate release of α-sarcin into the cytosol [3]. We likewise expect that the disulfide cross-linked VP7 mutant, which also has impaired infectivity, will not release DLPs, because it prevents loss of VP7 from the DLP surface [25].

Conventional thin-section electron microscopy of cells exposed to rotavirus particles shows at least three apparent morphological stages of viral uptake – particles bound at the cell surface, particles partly engulfed in a surface invagination, and particles apparently fully enclosed within a tightly surrounding vesicle. In all three cases, the distance between the densely stained center of the virion and the closest segment of the cell surface appears to be about 500–600 Å. For fully enclosed particles, the surrounding membrane vesicle is generally concentric with the virion. The cryoET analysis yields more accurate dimensions, without distortion from fixation and sectioning, and shows two distinct states of the virion-membrane contact. The distance from the center of the particle to the outer surface of the membrane is 480–500 Å for the “long” contacts – just as expected for sialic-acid binding by spikes in the conformation seen by single-particle analysis. The corresponding distance for the “short” contacts is about 410 Å.

The images suggest that the virus drives its own engulfment, by multiple contacts with the target-cell membrane. RRV binds sialic acid in a pocket on the outward-facing surface of VP8* [14], and the functional receptor on the cell surface is probably a sialylated ganglioside [19]. The distance from the center of a rotavirus particle to the sialic-acid binding sites on the outward facing surfaces of VP8* is about 480 Å, and the sialic acid of a ganglioside such as GD1a, at the outer tip of the glycan, can project 10–20 Å above the mean plane of phospholipid polar headgroups. Thus, the radial position of the receptor-binding site and the length of a typical glycolipid glycan account for the observed distance between the center of the virus particle and the surface of the membrane that contacts or surrounds it, and the micrographs are compatible with the notion that most or all of the VP8* lectin domains on a particle bind a glycosphingolipid sialic-acid group. At normal glycolipid compositions, a spherical shell of membrane 1000 Å in diameter will present several hundred glycans to an attached virus, more than enough to saturate the 120 VP8* domains at the interface.

Proposed non-ganglioside receptors for rotaviruses include integrins (αVβ3 for a site on VP7 and α2β1 for a site on VP4) [28]–[30] and Hsc70 [31]. We have explained elsewhere [6], [7] that the hypothesized attachment sequence on VP7 is buried at the VP7-VP6 contact, so any potential role for αVβ3 integrin would have to be at a post-uncoating step. The DGE sequence on VP5* suggested to be a site for α2β1 [30] is at the base of the protruding spike, near the surface of VP7, and so oriented that the Asp and Glu of DGE (the potential MIDAS-site interacting residues) face away from the surface of the subunit, both on the A and B subunits and on the C subunit [9]. Thus, like the VP7 site, access to α2β1 would probably require a post-uptake conformational change. Hsc70, which can bind almost any protein, is cytoplasmic; if small amounts of a related Hsp 70 were to appear on the cell surface, it would not be an abundant molecule. The uniformity of wrapping, shown by both cryoET and by conventional EM, and the direct contact of spikes with membrane, shown by cryoET, rule out any irregular, elongated, or low abundance receptor, at least for the engulfment step we have examined.

The short class of membrane contacts shown in Fig. 8 requires a conformational change in the spike. The relatively flexible tethering of the VP8* lectin domain to the “foot” of the spike through its extended, N-terminal linker [9] would enable VP8* to move away from the tips of VP5* without dissociating from the foot (Fig. 1C), exposing the VP5* hydrophobic loops. If these loops then insert into outer leaflet of the membrane bilayer, the observed separation agrees well with estimates from the structure. Tomogram sections such as the one in Fig. 8C suggest that this conformational change can occur progressively as the particle engulfs, potentially also explaining why a mutation in a VP5* hydrophobic loop extends the interval of lateral motion on the cell surface following initial attachment.

The current observations cannot rule out participation of cellular proteins in the stages of entry we have outlined, but they limit the requirements in the combination of virus strain and host cell we have studied. Electron microscopy shows occasional images of virions captured by a clathrin-coated pit or vesicle (Fig. 7). Although this route of uptake is clearly rare in BSC-1 cells, as we could not detect colocalization with AP-2 by live-cell imaging, it is reasonable to expect that in other cells – and for other rotavirus strains – a clathrin route might predominate [20], [32]. The tightness with which VP8* binds its ganglioside receptor, the abundance of the receptor on the cell surface, and the membrane tension (which affects the free energy of invagination) could all determine whether invagination needs assistance from the clathrin machinery. Clathrin coats disassemble within 10–15 secs of pinching off [33], [34], and the resulting uncoated vesicle resembles closely the virion-generated vesicles we find in our experiments; the diameter of the vesicle itself would also be about the same (Fig. 7B). Thus, release from vesicles derived from clathrin uncoating would have the same mechanism and potentially a similar time course as release from the vesicles detected in the experiments reported here.

Our finding, that in BSC-1 cells, RRV does not enter from Rab5 endosomes, appears at first to be at variance with published conclusions that the virus traffics to Rab5 comparments in MDCK cells [35] and MA104 cells [36]. In the former study, overexpressing the same Rab5 mutants used in our experiments gave only a twofold increase for constitutively active Rab5 and a twofold decrease for inactive Rab5; in the latter study, inactive Rab5 had roughly a fourfold effect, while constitutively active Rab5 had no effect at all. These relatively modest differences, compared with those of one or more orders of magnitude produced by mutations in VP5* [3] and VP7 [25], might reflect variation of principal entry routes in the cell types used, but they might instead reflect indirect effects of perturbations in membrane traffic under the conditions of cell growth in the different experiments. Linkage among pathways of membrane traffic makes inferring mechanism from indirect readout particularly challenging. For example, “knock-down” with siRNA of various endosome-associated proteins affects entry and infectivity (twofold to fourfold relative to cells transfected with irrelevant siRNA) [36], [37], but the cells have had several generations to adjust to the loss of function. We also detect virus in Rab5 endosomes, but those particles rarely uncoat and penetrate (Fig. 6). It is certainly possible that in other cells or under different conditions, virions might bud into endosomal membranes and penetrate from that compartment – for example, if the relevant glycan receptor for the strain in question were abundant on the endosomal luminal membrane. Nonetheless, the subsequent mechanism of membrane disruption, rupture of a small vesicle, would be the same as studied here.

The kinetics of entry we have analyzed agree well with earlier, less direct observations. Measurements of RRV internalization kinetics in MA104 cells, with protection from neutralization by mAb 159 as a readout, showed roughly 50% protection within 3–5 minutes of warming cells from 4°, at which virus attached but did not internalize, to 37°C [38]. Particles that had not been treated with trypsin attached efficiently, but internalized much more slowly than did trypsinized particles, with a half-time of 30–50 minutes. These observations suggest that entry into MA104 cells proceeds by stages similar to the ones we have followed in BSC-1 cells and that rapid, clathrin-independent internalization requires VP4 cleavage. There is ample trypsin in the part of the gut in which rotavirus propagates, and trypsin treatment of virions harvested from cell culture is probably a good surrogate for a normal, in vivo event.

The polyomaviruses, in particular SV40 and murine polyoma virus, have glycolipid receptors [39] and appear to enter at the cell surface by a process that closely resembles the “wrapping” we infer from images such as those in Fig. 7B [40], [41]. SV40 can induce tight-fitting invaginations even in ganglioside-containing, giant unilamellar vesicles with no cellular proteins, but scission, which presumably occurs during infectious entry, may require cellular factors in addition to the glycoplipids [41]. Dynamin recruitment appears not to be important for scission of the virus-containing invaginations and formation of the “autoendocytic vesicles” we describe here for rotavirus; whether other cellular proteins have any role remains an open question.

Even for penetration-incompetent particles (e.g., those bearing specific mutations in VP4 or VP7), the overall kinetics of sequestration from EDTA or antibody are the same as for particles recoated with wild-type proteins. Thus, the functions disrupted by the mutations – VP5* membrane interaction and VP7 dissociation – affect only the membrane-disrupting steps. Close inspection of the TLP structure [9] suggests that with VP7 in place, the spike could reorganize to allow the hydrophobic loops of all three VP5* subunits to engage the target membrane (step 1 in Fig. 1c), but that VP7 would hinder the folding back we postulate drives bilayer disruption (step 2). Loss of Ca^2+^ from the vesicle that encloses the virion is presumably the event that induces VP7 dissociation. Transient Ca^2+^ leaks in the membrane should allow the few thousand Ca^2+^ ions in a vesicle of the observed diameter (including those bound by VP7) to move rapidly down their concentration gradient into the cyotosol. Such leaks might be produced by the inserted VP5* loops, either by perturbations in the membrane bilayer from the loop insertion or by fluctuations of the VP5* trimer toward its folded-back conformation.

Our results bear on the molecular details of membrane disruption and DLP release. The critical observation is that release is from a relatively small vesicle that conforms closely to the outer diameter of the virion, not from a much larger endosome. A potential molecular consequence of this observation is illustrated by the right-hand panel in Fig. 1C. We have proposed previously that the conformational change in VP5* leading to the stable, folded-back structure generates the disruptive force that breaks the membrane [2]. The transition couples to the membrane through insertion of the hydrophobic loops at the tip of the β-barrel domain [3], [23]. A transition from an extended to a folded back structure will inevitably force the membrane to expand in area, because the local “bubble” created by any one trimer must be at the expense of membrane elsewhere. The bursting point for a lipid bilayer undergoing lateral expansion is about 3%. Distortion of the membrane as shown in Fig. 1C, even by a VP5* trimer released from its underlying DLP, will produce an approximately 0.5% expansion for each VP5* trimer that attempts to fold back, implying that membrane-coupled conformational reorganization of even a modest fraction of the 60 spikes on a virion will be enough to disrupt a small, tightly fitting vesicle as seen in the experiments reported here. A larger membrane-bound compartment, such as a Rab5 or Rab7 endosome, can withstand many more local impositions of sharp curvature, by compensating elsewhere in its extended and potentially pleated surface. Thus, if folding back of VP5* is indeed the mechanism of membrane disruption, DLP escape is very likely to be from a small, membrane vesicle, closely wrapped around the particle, rather than from a much larger one.

The picture we have acquired for uptake and penetration of a non-enveloped virus and its interpretation in molecular terms has depended both on detailed structural information from x-ray crystallography and cryoEM and on tracking of large numbers of individual particles by live-cell imaging. To confirm the proposed sequence of molecular events, we need to determine the stage at which the hydrophobic loops of VP5* engage the cellular membrane and the timing and location of the VP5* fold-back step, relative both to dissociation of VP7 and to release of the DLP. On-going enhancement of imaging sensitivity and use of context-dependent labeling (e.g., Ca^2+^ sensitive fluorophores) should make it possible to resolve these issues and thus to connect the molecular events sketched in Fig. 1c even more intimately with the cellular steps outlined in Figs. 2 and 7.

## Materials and Methods

### Cells

BSC-1 cells, and the derived cell line stably expressing α2-eGFP [42], were maintained at 37°C and 5% CO_2_ in DMEM (Invitrogen Corporation), supplemented with 10% fetal bovine serum (Hyclone Laboratories). To obtain cells expressing dynamin2-eGFP or Rab5-eGFP, approximately 60,000 BSC-1 cells were seeded into 6-well plates and transfected with 0.5 µg of plasmid encoding either rat dynamin2-eGFP (gift of Dr. Sandra Schmid) or 0.5 µg Rab5-eGFP (Addgene), with the aid of FUGENE 6, used according to the manufacturer's instructions (Roche Diagnostics). Cells were then trypsinized and re-seeded in T25 mL flasks (Corning) in the presence of G418 for at least 48 h, to select for cells that expressed the tagged protein at levels not detrimental to cell growth.

### TLP, DLP and recombinant protein purification

TLPs, DLPs, VP7, VP7 disulfide mutant, and m159 antibody were purified as previously described [7], [23], [25]. For TLP and DLP production, MA104 cells were grown in 10-stack cell-culture chambers (Corning), and confluent monolayers were infected with rhesus rotavirus (RRV; G3 serotype), at MOI of 0.1 focus-forming unit (FFU)/cell in M199 medium supplemented with 1 mg/mL porcine pancreatic trypsin (Worthington Biochemical). We collected the cell culture medium 24–36 h post infection, when cell adherence was <5%, and purified the TLPs and DLPs by freeze-thawing, ultracentrifuge pelleting, Freon-113 extraction, and cesium chloride gradient centrifugation. WT and C-C VP7 were expressed in Sf9 cells infected with a baculovirus vector and purified by successive affinity chromatography with concanavalin A and monoclonal antibody (mAb) 159, which is specific for VP7 trimer (elution by EDTA). DLPs and VP7 were dialyzed into amine-free buffers containing 25 mM Hepes pH 7.5 (VP7: 25 mM Hepes pH 7.5, 100 mM NaCl, 1 mM CaCl_2_; DLP: 25 mM Hepes pH 7.5, 100 mM NaCl, 0.1 mM EDTA). We expressed WT and V391D VP4 in baculovirus-infected insect cells [2]; the harvested cells were flash frozen and lysed by thawing; PMSF was added to 1 mM when thawing was complete. The lysate was clarified by centrifugation, and VP4 was precipitated by addition of AmSO_4_ to 30% saturation. The AmSO_4_ pellet was resolubilized in a volume of 25 mM Tris pH 8.0, 10 mM NaCl, 1 mM EDTA that gave a conductance matching that of Phenyl HP Start Buffer (25 mM Tris pH 8.0, 3.5M NaCl, 1 mM EDTA) and loaded onto a Phenyl HP column (GE Healthcare). Following elution with 25 mM Tris pH 8.0, 10 mM NaCl, 1 mM EDTA, fractions containing VP4 were pooled, dialyzed against the same buffer, loaded onto a HiTrap Q column (GE Healthcare), and eluted in Phenyl HP Start Buffer. Pooled fractions containing VP4 were then concentrated to 1–2 mL with a Centriprep 50 concentrator (Millipore) and subjected to a final purification on S200 (GE Healthcare) in 25 mM Hepes pH 7.5, 100 mM NaCl, 0.1 mM EDTA.

### Dye conjugation to DLPs and proteins

DLPs, VP7, VP4 and m159 antibody in amine-free buffer were conjugated to amine-specific Atto dyes (ATTO-TEC) as follows. NaHCO_3_ (pH 8.3) was added separately to each of the components listed to a final concentration of 0.1 M, and Atto dyes, suspended in anhydrous DMSO (Sigma) to 2 mg/mL, were then added to obtain the following final dye concentrations: for DLP labeling, 20 µg/mL; VP4, 16 µg/mL; VP7, 20 µg/mL; m159, 50 µg/mL. For recoated particles, dye combinations were varied according to the objectives of the experiment (e.g., doubly vs. triply labeled particles). DLPs. Proteins were incubated with dye for 1 h in the dark at room temperature, and the reactions were quenched by adding Tris pH 8.0 to a final concentration of 200 mM. Labeled components were then dialyzed into buffers for ensuing recoating reactions (see below for recoating methods). For recoated particles, dye combinations were varied according to the objectives of the experiment (e.g., doubly vs. triply labeled particles).

### Recoating

Recoating was carried out as previously described [22], [23], using recoating components labeled as described above. Briefly, Atto-labeled, recombinant VP4 was added to purified DLPs in at least 5-fold excess in buffer adjusted to pH 5.2 with sodium acetate, and the pH of the mix was adjusted to pH 5.2 by stepwise addition of sodium acetate and testing by pH paper. After incubation at room temperature for 1.5 h, mutant or WT VP7 was added in at least 3-fold molar excess in buffer supplemented with calcium, and the mixture was incubated for a further 30 minutes at room temperature. Recoated particles were separated from excess labeled components by cesium chloride gradient centrifugation and dialyzed into appropriate buffers (25 mM Hepes, 100 mM NaCl and 1 mM CaCl_2_). Titers of recoating reactions were determined by infectious focus assays as described [25].

### Neuraminidase treatment

BSC-1 cells, plated on 25 mm No. 1.5 coverslips at a density of 150,000 cells/coverslip, were grown overnight at 37°C. The cells were washed twice with HEPES pH 7.0, 140 mM NaCl, 1 mM CaCl_2_; 300 µl of α-MEM supplemented with 1 mM CaCl_2_, with or without 100 mU/ml *Vibrio cholera* neuraminidase (Sigma), was then added and the plates incubated for 1 hr. at 37°C. Recoated TLPs (RcTLPs), prepared with Atto 565 labeled VP7, unlabeled VP4, and Atto 647N labeled DLPs, were activated by 1∶10 dilution in 5 µg/ml trypsin in TNC buffer at 37°C for 30 min and then placed on ice until use. Trypsin-treated RcTLPs (30 µl, added to 70 ul of α-MEM and mixed) were added directly to the medium on the plates (final MOI∼15). After 15 min of incubation of cells and RcTLPs, confocal z-stack images were collected using transmitted light (cell) or laser excitation at 561 nm (VP7 Atto 565). For focus-forming assays, confluent BSC-1 monolayers were incubated for 1 hr. at 37°C in α-MEM supplemented with 1 mM CaCl_2_, with or without 100 mU/ml *V. cholera* neuraminidase. RcTLPs or native TLPs were then added at an MOI of 15 and allowed to bind at 4°C for 2 hrs. Following incubation, cells were washed three times in PBS, freeze-thawed three times, and the amount of infectious virus bound determined by focus-forming assay on fresh, confluent BSC-1 cells.

### Live cell imaging and data analysis

Approximately 1×10^5^ BSC-1 cells were grown on 25 mm No. 1.5 coverslips as described above. Medium was exchanged immediately before imaging with pre-warmed MEM–α, without phenol red, supplemented with 25 mM Hepes (pH 7.4) and 2% FBS (Hyclone). Labeled recoated virus particles were added to cells at MOI of 0.1–0.2. For experiments in Figure 2, images were acquired every 1 minute (Fig. 2D) or 1 s (Fig. 2E,F) using 100 ms (Fig. 2D) or 5–30 ms (Fig. 2E, F) exposure times (no binning) with a previously described laser and confocal microscope configuration [43]. Image and data analysis was performed using Slidebook 4.2 (Intelligent Imaging Innovations, Denver CO). For experiments in Figures 3–6, cells were grown on No. 1.5 coverslips as described above and mounted on a Prior Proscan II motorized stage on a Nikon Ti inverted microscope equipped with 100× Plan Apo NA 1.4 objective lens and the Perfect Focus System. The microscope was enclosed in a custom built, heated chamber warmed to 37°C; the sample was supplied with 5% CO_2_. All images were collected with a Yokagawa CSU-X1 spinning disk confocal with Spectral Applied Research Borealis modification Excitation with solid state lasers was controlled by an AOTF; images, acquired with a Hamamatsu ORCA-AG cooled CCD camera controlled by MetaMorph 7 software, were collected with a 405/491/561/642 band pass dichroic mirror (Semrock) at the following wavelengths: 491 nm line with a 525/50 emission filter; 561 nm line with a 620/60 emission filter; 642 nm with a 700/75 emission filter (Chroma). For time-lapse experiments, images were collected every 3–6 s depending on the objectives of the experiment (described in respective figure legends), using an exposure time of 5–30 ms and 2×2 binning, with illumination attenuated by the AOTF between acquisitions. Gamma, brightness, and contrast were adjusted on displayed images (identically for compared image sets) using MetaMorph 7 software. Analysis was performed using built-in functions provided by Slidebook 4.2 and MetaMorph 7. EDTA flow-in experiments were performed during imaging experiments without interruption of image collection by quickly pipetting away the MEM-a/FBS medium bathing the cells and replacing the medium by gently layering pre-warmed MEM-a/FBS medium containing 4 mM EDTA onto the culture plate; we were careful not to disturb the plane of focus. Although EDTA treatment caused cells ultimately to detach from the coverslip, they remained in place long enough to determine whether a bound virus particle resisted dissociation. m159 flow-in experiments were performed similarly; the replacement medium contained 25–50 µg/mL fluorescently labeled m159 antibody rather than EDTA.

### Assay for Rab5 effects

BSC-1 cells, seeded in complete growth medium (DMEM supplemented with Pen/Strep and 10% FBS) at 70% confluence on 6-well plates, were transfected with plasmids expressing GFP-Rab5DN(S34N) and GFP-Rab5CA(Q79L) as described [44]. After 24 h at 37°C, the cells were trypsinized and replated at ∼30% confluency on polylysine-coated glass coverslips placed in 6-well plates. After another 24 h at 37°C, cells were washed twice with warmed phosphate buffered saline (PBS), and RRV TLPs, freshly treated with trypsin in α-MEM, were added to each well at the indicated MOI. Cells were incubated with virus at 37°C for 1 hr, after which the medium was replaced with DMEM supplemented with Pen/Strep and neutralizing mAb m159 at 3.6 µg/ml, and the infection was allowed to proceed for 16–18 hours at 37°C. After washing the cells with serum-free α-MEM at 37°C, transferrin labeled with Alexa-Fluor 647 (Tf-647) was added at 50 µg/ml. The cells were incubated with the Tf-647 for 10 min at 37°C, washed with PBS, fixed for 10 min with 3.7% formaldehyde in PBS, and prepared for immunofluorescence as described [22]. Infection was assayed by staining with anti-VP7 mAb m60 and counterstaining with Alexa Fluor 568 labeled goat anti-mouse IgG. Images were acquired with a Mariana system (Intelligent Imaging Innovations, Denver, CO) based on a Zeiss AxioVert 200M inverted microscope (Carl Zeiss Microimaging, Inc., Thornwood, NY) equipped with a CSU-22 spinning-disk confocal unit (Yokogawa Electric, Tokyo, Japan), a piezo-driven Z-translation, and linear encoded X&Y translations and controlled with SlideBook V5.0 (Intelligent Imaging Inc., Denver, CO). Excitation wavelengths were 491, 561, and 660 nm (lasers from Cobolt, Solna, Sweden); the emission filters ranges were 525–550 nm, 620–660 nm, and 680 nm long-pass (Semrock, Rochester, NY). We collected Z-scans at 10–12 positions in each sample, imaging the entire cell volume in 0.5 micron steps, with exposure times per step of 30 ms at 491 nm (GFP-Rab5) and 50 ms at 561 nm (Alexa-Fluor 568 goat anti-mouse IgG) and 660 nm (Tf-647). For each field of view (10–12 fields/experimental condition), we scored cells for Rab5 expression, RRV infection, and Tf uptake, the last as a control for clathrin-based receptor mediated endocytosis. As each coverslip had transfected and non-transfected cells, we could score both expressing and non-expressing cells for RRV infection in the same fields.

### Thin-section electron microscopy

Authentic, trypsin-treated TLPs were added at 280 ffu/cell to BSC-1 cells that had been pre-incubated with MEM-a (without FBS) for 10 min. The cells were fixed 5–10 min after adding virus by incubating for 1 hr in fixative (1.25% formaldehyde, 2.5% glutaraldehyde, 0.03% pictric acid in 0.1 M sodium cacodylate, pH 7.4). Fixed cells were stained successively with osmium tetroxide (1%) and uranyl acetate (1%), washed, dehydrated with successive washes in 75%, 90% and 100% ethanol, soaked in propyleneoxide for 1 hr, and infiltrated and embedded in Epon (polymerized for 24–48 hr at 60°C). Sections about 50 nm thick were examined in the FEI Tecnai G2 Spirit BioTWIN electron microscope of the Harvard Medical School Electron Microscopy Core Facility.

### CryoET

Freshly glow-discharged EM gold-grids coated with a holey carbon film (Quantifoil R 2/2 200 mesh, Quantifoil MicroTools GmbH, Germany) were set on the bottom of a sterile Petri dish and sterilized with 70% ethanol for 10 minutes. The sterilized grids were rinsed twice with 0.2 µm filtered water, submerged in 0.1% poly-l lysine hydrobromide overnight, and then rinsed once in 0.2 µm filtered water and twice in unsupplemented MEMα media. BSC-1 cells were plated over 6 prepared grids at a density of 9×10^4^ cells/ml in a total of 2 ml MEMα supplemented with 10% FBS in a 35 mm diameter glass bottom dish (MatTek). The cells were incubated for 24 hrs. at 37 deg. C and 5% CO_2_. They attached to the grids at low density (about one cell per three grid squares), which we verified by DIC light microscopy. Grids with cultured host cells, held by self-closing tweezers at the edge of the grid, were washed with three drops of medium, and TLPs at 9.4×10^9^ FFU/ml in 5–10 µl were added to the cells on the grid. The cells were incubated at 37°C for 30 min, after which 1.5 µl of 10-nm colloidal gold solution (Sigma-Aldrich, St. Louis, MO) was added to create fiducial markers for use during tilt series alignment and tomogram reconstruction. Excess fluid was blotted with filter paper just before rapid freezing by plunging into liquid ethane, using a manual plunge freezing device. The frozen grids were stored in liquid nitrogen.

For imaging, we used a Tecnai F30 transmission electron microscope (FEI, Inc., Hillsboro, OR) operating at an accelerating voltage of 300 kV. The microscope was equipped with a field emission gun, a high-tilt stage, a post-column energy filter (Gatan Inc., Pleasanton, CA) and a 2k×2k charge-coupled device camera (Gatan). We recorded low-dose images at −8 µm defocus and at a nominal magnification of 13,500×, giving a pixel size of 9.86 Å. Single-axis tilt series were recorded by tilting the specimen from −60° to +60° in 1.5–2° increments using SerialEM control software [45]; the total electron dose per tilt series was kept below 150 e/Å^2^.

We used IMOD [46] for fiducial alignment of the tilt series images and for tomogram reconstruction by weighted back-projection. Virus particles were picked from the raw 3D cryo-tomograms. Bound and unbound particles were identified by visual inspection. Subtomogram averaging was performed with PEET [47], using the published cryo-EM structure (EMDB 5199) [9], filtered to 25 Å resolution, as an alignment reference. Icosahedral symmetry was applied as described by the Boulder Laboratory for 3D Electron Microscopy of Cells online protocol (http://www.youtube.com/watch?v=c9LqABmRd7Q&list=PLGggUwWmzvs_Q8j05yw2B2vVstVZaT9at). We averaged 18 unattached particles (18×60 = 1080 repeats), and 7 membrane-attached particles (7×60 = 420 repeats). For the class of virons bound to the host cell membrane, we also calculated a “membrane-preserving” average using only those repeats that contained spikes in contact with the membrane (78 repeats from 5 particles). We used IMOD for 3D visualization and isosurface rendering of the averaged particles.

## Supporting Information

Figure S1 **Effect of neuraminidase pre-treatment of BSC-1 cells on rotavirus attachment and infectivity**. **A.** Attachment. BSC-1 cells, plated on coverslips as described in Methods, were treated for 1 hr at 37°C with 100 mU/mL *Vibrio cholerae* neuraminidase. Recoated particles, labeled on VP7 with Atto565 and activated with trypsin as described in Methods, were then added, and spinning-disk confocal z-stacks recorded after 15 min incubation. The figure shows maximum intensity projections. Upper row: control; lower row: neuraminidase treated. Left-hand panels, transmitted-light images of a cell in the field; middle panels, fluorescence images (laser excitation at 561 nm) from the same cell; right-hand panels, overlay of the other two. The white curve shows the edge of the cell; neuraminidase treatment does not affect adsorption of particles to the coverslip. **B.** Infectivity. Confluent BSC-1 monolayers were incubated for 1 hr. at 37°C with or without 100 mU/ml *V. cholera* neuraminidase. RcTLPs or native TLPs were then added and allowed to bind at 4°C for 2 hrs. Cells were washed and freeze-thawed, and the amount of infectious virus bound determined by focus-forming assay (see Methods) on fresh, confluent BSC-1 cells. The reduction to about 20% of the infectivity on untreated cells is comparable to most published measurements (see [49], for example). The residual attachment seen in the central panel of the upper row, is probably due to a combination of incomplete elimination of terminal sialic acids and on-going insertion into the membrane of newly synthesized sialylated glycolipids.
(TIF)

Figure S2 **Lateral motion on cell surface of attached wt, VP4 fusion-loop mutant, and VP7 C-C mutant particles.** **A**. Tracks of particles, imaged at 3-sec intervals, immediately after addition to BSC-1 cells. Total tracking time: 3 mins. **B**. Lateral-motion time for 100 individual particles recoated with the VP4 fusion-loop mutant, with average and median. **C**. Lateral-motion time for 100 individual particles recoated with VP7 C-C, with average and median.
(TIF)

Figure S3 **Effects of ectopic expression of Rab5 mutants on rotavirus infectivity.** BSC-1 cells were transfected with plasmids encoding GFP-Rab5CA(Q79L) or GFP-Rab5DN(S34N), as described in Materials and Methods, plated after 24 hr onto glass coverslips, and infected 24 hr later with RRV at the indicated multiplicity of infection (moi). Each coverslip had transfected and untransfected cells (GFP positive and negative Rab5 endosomes, respectively); the latter give an internal control in the same field as the transfected cells. In each panel, the bar chart shows the percent of cells infected for GFP positive (blue) and GFP negative (red), with the number of cells counted and the number infected shown as ratios above each pair of bars. The data shown are from two completely independent experiments on different days. **A.** Constitutively active, Rab5CA. **B.** Dominant negative, Rab5DN.
(TIF)

Figure S4 **Effect of hydroxy-dynasore on rotavirus entry.** **A**. Effect of adding inhibitor after adding virus. BSC-1 cells stably expressing σ2-adaptin fused to EGFP (σ2-EGFP) were washed twice with FBS-free α-MEM before infection with RRV at 37°C for the indicated times. The cells were then washed twice with α-MEM and further incubated with medium containing 1∶2000 m159 monoclonal neutralizing antibody as well as 0.5% DMSO or 20 µM hydroxy-dynasore (Sigma) for ten minutes. The cells were then kept overnight at 37°C in α-MEM containing 10% FBS, 1% penicillin streptomycin, and 1∶2000 m159 and fixed the following day with methanol. Infectious foci were detected by immunoperoxidase staining, using the monoclonal antibody, M60, as the primary detection antibody. Error bars represent triplicate titrations of each time point. **B**. Inhibition of Tf uptake, hydroxy-dynasore added with Tf. BSC-1 cells stably expressing σ2-EGFP were incubated with α-MEM containing 0.5% DMSO or 20 µM hydroxy-dyanasore for ten minutes at 37°C. The media were then replaced, respectively, with ones containing 0.5% DMSO; 0.5% DMSO and 10 µg/ml transferrin fluorescently labeled with Alexa 647 (Tf647); or 20 µM hydroxy-dyanasore and 10 µg/ml Tf647 for ten minutes at 37°C. Samples were then acid washed before fixation. **C**. Effect of adding inhibitor before adding virus. BSC-1 cells stably expressing σ2-EGFP were washed twice with FBS free α-MEM and incubated for ten minutes in media containing 0.5% DMSO carrier or 20 µM hydroxy-dynasore. The cells were washed twice with α-MEM before infection with RRV for twenty minutes at 37°C. The cells were then incubated overnight at 37°C in α-MEM containing 10% FBS, 1% penicillin streptomycin, and 1∶2000 m159, fixed with methanol the following day, and infectious foci were detected as in A. Error bars represent triplicate titrations of each condition. **D**. Inhibition of Tf uptake, hydroxy-dynasore added before Tf. BSC-1 cells stably expressing σ2-EGFP were incubated with α-MEM containing 0.5% DMSO or 20 µM hydroxy-dynasore for ten minutes at 37°C. The cells were then washed twice with α-MEM and then incubated in media containing 0.5% DMSO or 0.5% DMSO and 10 µg/ml Tf647 for twenty minutes at 37°C. Samples were then acid washed before fixation.
(TIF)
