# Supplementary figures and images for: Structural Correlates of Rotavirus Cell Entry
Source: PLoS Pathog. 2014 Sep 11;10(9):e1004355. doi: 10.1371/journal.ppat.1004355 (PMC4161437; doi:10.1371/journal.ppat.1004355)

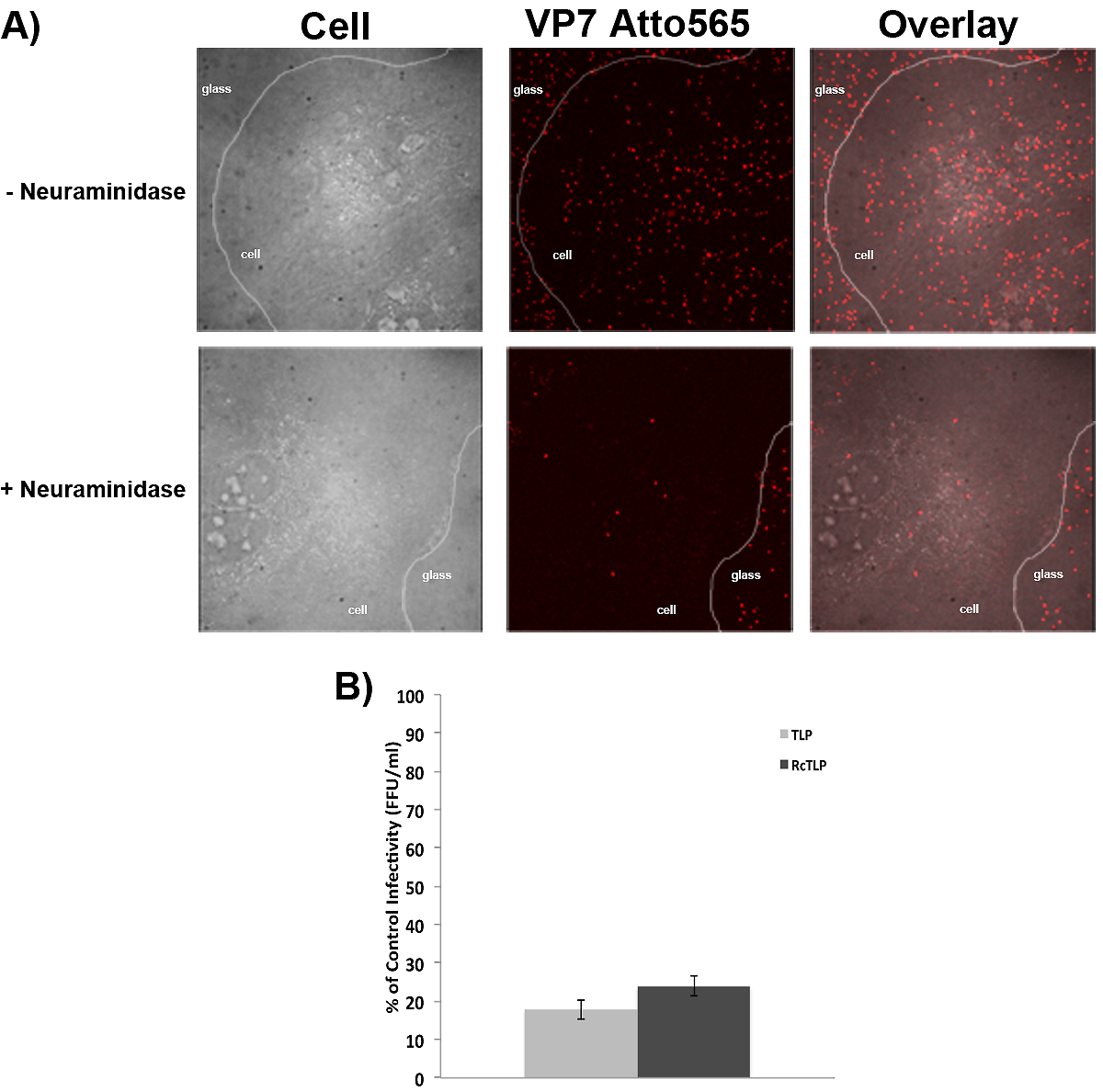

Supplement: Figure S1 — Effect of neuraminidase pre-treatment of BSC-1 cells on rotavirus attachment and infectivity. A. Attachment. BSC-1 cells, plated on coverslips as described in Methods, were treated for 1 hr at 37°C with 100 mU/mL Vibrio cholerae neuraminidase. Recoated particles, labeled on VP7 with Atto565 and activated with trypsin as described in Methods, were then added, and spinning-disk confocal z-stacks recorded after 15 min incubation. The figure shows maximum intensity projections. Upper row: control; lower row: neuraminidase treated. Left-hand panels, transmitted-light images of a cell in the field; middle panels, fluorescence images (laser excitation at 561 nm) from the same cell; right-hand panels, overlay of the other two. The white curve shows the edge of the cell; neuraminidase treatment does not affect adsorption of particles to the coverslip. B. Infectivity. Confluent BSC-1 monolayers were incubated for 1 hr. at 37°C with or without 100 mU/ml V. cholera neuraminidase. RcTLPs or native TLPs were then added and allowed to bind at 4°C for 2 hrs. Cells were washed and freeze-thawed, and the amount of infectious virus bound determined by focus-forming assay (see Methods) on fresh, confluent BSC-1 cells. The reduction to about 20% of the infectivity on untreated cells is comparable to most published measurements (see [49], for example). The residual attachment seen in the central panel of the upper row, is probably due to a combination of incomplete elimination of terminal sialic acids and on-going insertion into the membrane of newly synthesized sialylated glycolipids. (TIF) [file ppat.1004355.s001.tif]

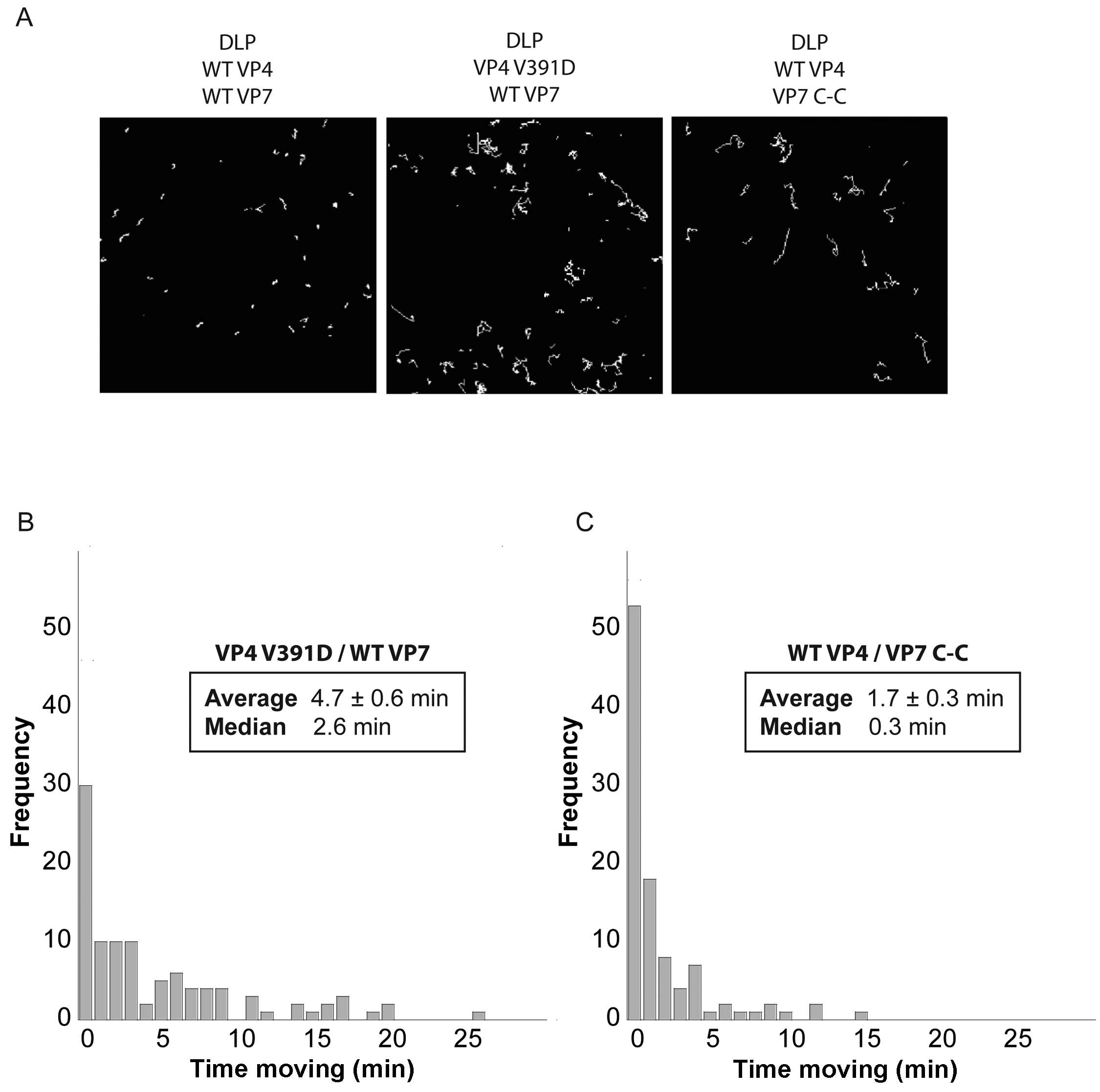

Supplement: Figure S2 — Lateral motion on cell surface of attached wt, VP4 fusion-loop mutant, and VP7 C-C mutant particles. A. Tracks of particles, imaged at 3-sec intervals, immediately after addition to BSC-1 cells. Total tracking time: 3 mins. B. Lateral-motion time for 100 individual particles recoated with the VP4 fusion-loop mutant, with average and median. C. Lateral-motion time for 100 individual particles recoated with VP7 C-C, with average and median. (TIF) [file ppat.1004355.s002.tif]

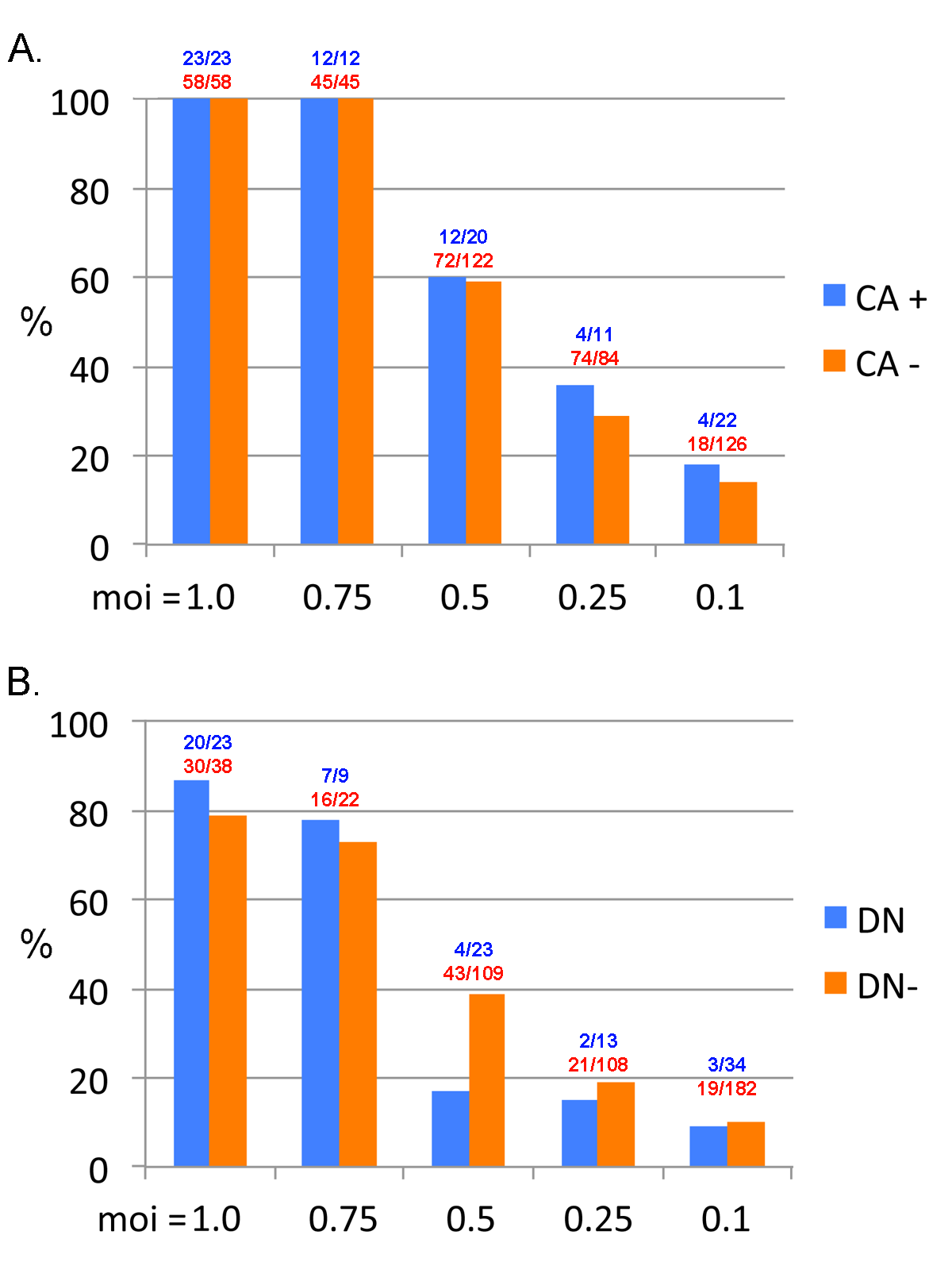

Supplement: Figure S3 — Effects of ectopic expression of Rab5 mutants on rotavirus infectivity. BSC-1 cells were transfected with plasmids encoding GFP-Rab5CA(Q79L) or GFP-Rab5DN(S34N), as described in Materials and Methods, plated after 24 hr onto glass coverslips, and infected 24 hr later with RRV at the indicated multiplicity of infection (moi). Each coverslip had transfected and untransfected cells (GFP positive and negative Rab5 endosomes, respectively); the latter give an internal control in the same field as the transfected cells. In each panel, the bar chart shows the percent of cells infected for GFP positive (blue) and GFP negative (red), with the number of cells counted and the number infected shown as ratios above each pair of bars. The data shown are from two completely independent experiments on different days. A. Constitutively active, Rab5CA. B. Dominant negative, Rab5DN. (TIF) [file ppat.1004355.s003.tif]

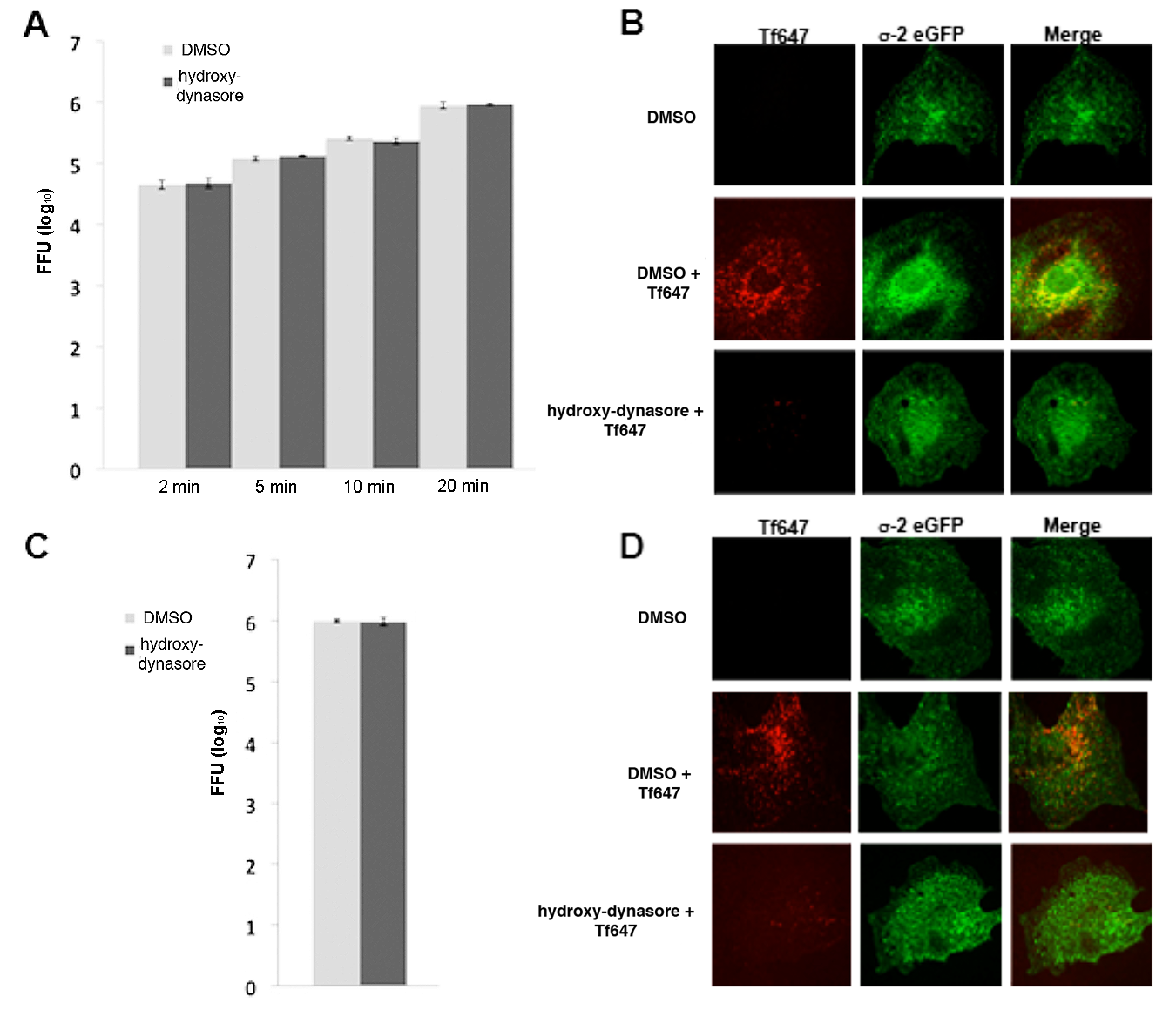

Supplement: Figure S4 — Effect of hydroxy-dynasore on rotavirus entry. A. Effect of adding inhibitor after adding virus. BSC-1 cells stably expressing σ2-adaptin fused to EGFP (σ2-EGFP) were washed twice with FBS-free α-MEM before infection with RRV at 37°C for the indicated times. The cells were then washed twice with α-MEM and further incubated with medium containing 1∶2000 m159 monoclonal neutralizing antibody as well as 0.5% DMSO or 20 µM hydroxy-dynasore (Sigma) for ten minutes. The cells were then kept overnight at 37°C in α-MEM containing 10% FBS, 1% penicillin streptomycin, and 1∶2000 m159 and fixed the following day with methanol. Infectious foci were detected by immunoperoxidase staining, using the monoclonal antibody, M60, as the primary detection antibody. Error bars represent triplicate titrations of each time point. B. Inhibition of Tf uptake, hydroxy-dynasore added with Tf. BSC-1 cells stably expressing σ2-EGFP were incubated with α-MEM containing 0.5% DMSO or 20 µM hydroxy-dyanasore for ten minutes at 37°C. The media were then replaced, respectively, with ones containing 0.5% DMSO; 0.5% DMSO and 10 µg/ml transferrin fluorescently labeled with Alexa 647 (Tf647); or 20 µM hydroxy-dyanasore and 10 µg/ml Tf647 for ten minutes at 37°C. Samples were then acid washed before fixation. C. Effect of adding inhibitor before adding virus. BSC-1 cells stably expressing σ2-EGFP were washed twice with FBS free α-MEM and incubated for ten minutes in media containing 0.5% DMSO carrier or 20 µM hydroxy-dynasore. The cells were washed twice with α-MEM before infection with RRV for twenty minutes at 37°C. The cells were then incubated overnight at 37°C in α-MEM containing 10% FBS, 1% penicillin streptomycin, and 1∶2000 m159, fixed with methanol the following day, and infectious foci were detected as in A. Error bars represent triplicate titrations of each condition. D. Inhibition of Tf uptake, hydroxy-dynasore added before Tf. BSC-1 cells stably expressing σ2-EGFP were incubated with α-MEM con [file ppat.1004355.s004.tif]
